# Preparation and evaluation of hair growth promoting effect of transferosomes containing red clover extract and caffeine alone or in combination

**DOI:** 10.22038/AJP.2024.24304

**Published:** 2024

**Authors:** Valiollah Hajhashemi, Azade Taheri, Farnaz Karimian, Omid Hajihashemi, Ardeshir Talebi

**Affiliations:** 1 *Department of Pharmacology and Toxicology, School of Pharmacy and Pharmaceutical Sciences, Isfahan University of Medical Sciences, Isfahan, Iran*; 2 *Department of Pharmaceutics, School of Pharmacy and Pharmaceutical Sciences, Isfahan University of Medical Sciences, Isfahan, Iran*; 3 *Department of Clinical Pharmacy, School of Pharmacy and Pharmaceutical Sciences, Isfahan University of Medical Sciences, Isfahan, Iran*; 4 *Department of Clinical Pathology, School of Medicine, Isfahan University of Medical Sciences, Isfahan, Iran*

**Keywords:** Mice, Minoxidil, Red clover, Transferosome, Trifolium pratense

## Abstract

**Objective:**

*Trifolium pratense* L. (Red clover) belongs to the Leguminosae family. This study was designed to develop transferosome formulations containing red clover extract or caffeine alone or in combination and evaluate their effects on hair growth in mice.

**Materials and Methods:**

Thin-lipid film hydration technique was used to make transferosomes. Six groups (n=6) of male Swiss mice (28-32 g) were used. One group was normal control. The second group received transferosome without drug. Groups 3 to 5 received 100 µl of transferosomes containing red clover extract (1%) or caffeine (0.002%) alone or in combination. The sixth group received minoxidil (2%). Treatments continued six days per week for 3 weeks and each week, the hair growth scores were recorded. At the end, sections of the skin were prepared for determining the percent of follicles in the anagen phase.

**Results:**

Encapsulation efficiency was 84.3, 81.6 and 89.1% for red clover, caffeine and red clover+caffeine transferosomes respectively. After 24 hr, the cumulative release of red clover and caffeine formulations was 77.6 and 76.9%, respectively. Treatments produced no significant change in hair growth after two weeks but at the end of the third week, all treatments significantly increased the hair growth and the effects were comparable with minoxidil. The combination of red clover and caffeine was not more effective than either alone.

**Conclusion:**

Transferosome formulations of caffeine and red clover alone demonstrated hair growth effect but their combination had no additive effect which might be due to a physicochemical or pharmacodynamic interaction.

## Introduction

Hair loss is a common problem that affects up to 50% of men and women in their lifetime. This problem can occur in any part of the body, but in cases where it only affects the scalp, it causes concern for the patient and an adverse effect on beauty (Faruk, 2018; Mounsey and Reed, 2009). In most cases of androgenic alopecia, dihydrotestosterone (DHT) is the main cause of hair loss (Lolli et al., 2017). 

Alopecia can be treated with a variety of agents, including minoxidil, caffeine, and 5-alpha reductase inhibitors, which can be given systemically or applied topically (Phillips et al., 2017; York et al., 2020). Adverse drug effects limit systemic administration, while topical formulations present difficulties in achieving effective drug delivery (Prausnitz and Langer, 2008). The stratum corneum, the outer layer of skin, acts as a major barrier to prevent drug absorption and creation of therapeutic concentrations in these systems (Menon et al., 2012). A significant body of research has been undertaken to overcome the limited permeability of this layer in order to boost medication concentration in hair follicles (Ruela et al., 2016). 

In recent years, many herbs and their metabolites have been tested for their hair growth effect. Caffeine is one of the secondary metabolites in more than 60 plant species and is also synthetically synthesized. Caffeine is one of the known components in coffee, green tea and black tea. Caffeine is present in many medicinal products to relieve muscle pain and headache, to treat menstrual symptoms and to prevent hair fall (Ohyama, 2021; Völker et al., 2020).

Caffeine inhibits 5-α-reductase, the enzyme responsible for converting testosterone to DHT. Caffeine also inhibits phosphodiesterase (PDE), the enzyme responsible for cAMP degradation. This increases intracellular levels of cAMP and stimulates cellular metabolism. In addition, caffeine causes blood vessels to dilate and thus increases blood supply to the follicles (Fischer et al., 2007).

*Trifolium pratense* L. (red clover) belongs to the Leguminosae family. This plant contains high amounts of isoflavonoids, compounds that are widely distributed in the Leguminosae family. The main isoflavones in red clover are biochanin A and formononetin (Kaurinovic et al., 2012).

Biochanin A is a nutritional supplement available for women suffering from menopausal symptoms. Isoflavones are structurally similar to estrogen, and exhibit agonistic and antagonistic interactions with the estrogen receptor. Biochanin A has antioxidant, anti-proliferative, and anti-inflammatory activity, protects dopaminergic nerve cells, stimulates osteoblastic differentiation and inhibits melanogenesis. It is also a strong inhibitor of 5-α-reductase, thus, it can be beneficial in male hair loss (Sundaresan et al., 2018; Wang et al., 2008).

Formononetin is an O-methylated isoflavone. Given that the structure of formononetin is relatively similar to endogenous estrogen (estradiol), formononetin is known as a phytoestrogen that is able to bind to estrogen receptors, namely estrogen receptors α and β. Formononetin causes vasodilation through endothelium-independent pathway and endothelium-dependent pathway with nitric oxide release (Ong et al., 2019; Sun et al., 2011; Tay et al., 2019).

Transferosomes are elastic or deformable vesicles which are applied to the skin in a non-blocking manner and have been shown to penetrate the skin through the lipid layers of the stratum corneum as a result of hydration or osmotic force (Kumar and Pradhan, 2022; Ramezani et al., 2018; Solanki et al., 2016). They are known as a successful tool for transdermal delivery for many drugs including large molecules, nucleic acids, proteins, and peptides as well as hydrophilic drugs. Unlike liposomes, they show more flexibility and do not have the limitations of lipophilicity and size of the therapeutic agents (Rajan et al., 2011). Presence of surfactants in transferosomes enhances drug penetration through the skin and increases the concentration of the drug in the deeper layers of the skin even in the blood circulation (Chauhan et al., 2017). Therefore, transferosomes are suitable delivery systems for compounds with low water solubility or skin penetration (Chauhan et al., 2017; Ramezani et al., 2018). 

According to the above mentioned points, the aim of this study was to prepare transferosomes containing red clover extract or caffeine alone and in combination, to evaluate their effects on hair growth in mice and to compare them with the standard drug minoxidil.

## Materials and Methods

### Materials

Lecithin, polysorbate 80 and polysorbate 20 were purchased from Merck Chemical Company (Germany). Caffeine was a gift from Farabi Pharmaceutical Company (Isfahan, Iran) and hydro-alcoholic extract of red clover was provided by Faraz Daru Company (Isfahan, Iran). The yield value of the extract was 11.2% and its total phenols was 9.69 mg/ml. Also, from 100 ml of the extract, 2.86 g freeze-dried powder was obtained.

### Transferosomes preparation

Thin-lipid film hydration technique was used to make transferosomes. First, 300 mg of lecithin, 22 mg of polysorbate 80 and about 10 mg of polysorbate 20 were weighed and dissolved in 6 ml of chloroform. Then, the obtained solution was transferred to a round bottom flask and the flask was connected to a vacuum dryer.

To make transferosomes containing red clover, 100 mg of red clover extract was added to above-mentioned formulation. Then, 10 ml of phosphate buffered saline (PBS) containing caffeine was added to the formed thin lipid layer and using glass balls, we allowed the aqueous solution containing PBS and caffeine to be placed in the vicinity of the lipid film and the lipid film hydrate and form transferosomes (Qushawy et al., 2018).

### Determination of entrapment efficiency

One milliliter of each formulation was centrifuged at 10000 rpm for 2 min. The supernatant was removed and the drug concentration was measured spectrophotometrically. The absorbance for caffeine was read at 274 nm. The absorbance of the red clover extract was tested in different wavelengths and its λmax was determined at 208 nm. Entrapment efficiency was calculated using the following formula: 

EE%=(C_t_-C_f_)/C_t_*100. 

C_t_: total concentration. C_f_: concentration of free drug

### Ex-vivo release of drugs

One milliliter of each formulation was transferred to a dialysis bag. A phosphate buffer (pH 6.3) was used as release medium. Samples were collected at 1, 2, 3, 4, 5 and 24 hr intervals and again drug concentration was measured spectrophotometrically as indicated in the previous section (Yu et al., 2019).

### Pharmacological experiments

Thirty-six male Swiss mice weighing 28 to 32 grams were used in groups of 6. The hair on the back of the animals was removed in an area of ​​2 x 4 cm using a depilatory cream. The animals received 100 microliters of the provided materials daily. This continued six days a week for 3 weeks. At the end of each week, hair growth was scored based on the scoring method (Hajhashemi et al., 2019). In this way, a score of 0 to 5 was considered for growth (0 for no hair growth, 1 for less than 20% growth, 2 for between 20 to 40%, 3 for 40 to 60%, 4 for 60 to 80% and 5 for 80 to 100 percent). On the 21st day, animals were sacrificed in a chamber containing carbon dioxide. The skin (1 X 1 cm) was cut and fixed in 10% formalin for pathological examination. All animal experiments were performed according to guidelines for the care and use of laboratory animals provided by the National Ethical Committee (Iran) (Ethics code: IR.MUI.RESEARCH.REC.1400.393). 

### Grouping of animals

 Animals were divided in six groups (n=6) and they received the following treatments: 1) Control group without treatment. 2) Transferosome without drug (carrier). 3) Transferosome containing 1% red clover extract. 4) Transferosome containing 0.002% caffeine. 5) Transferosome containing 1% red clover extract and 0.002% caffeine. 6) Minoxidil 2% (Bansal et al., 2012; Manzoureh and Farahpour, 2021) 

All animals received 100 µl of above drugs or carrier six days per week and treatments continued for 3 weeks (Hajhashemi et al., 2019).

### Statistical analysis

Parametric data was analyzed by one-way ANOVA followed by Scheffe post hoc, and non-parametric data was analyzed by Mann-Whitney U test. SPSS software (version 20) was used for data analysis and Excel 2020 was used to draw Figures.

## Results

### Entrapment efficiency

The entrapment efficiency for transferosome formulations is summarized in Table 1; all formulations had an entrapment efficiency of more than 80%.

### Ex vivo release of drugs

**Figure 1:**
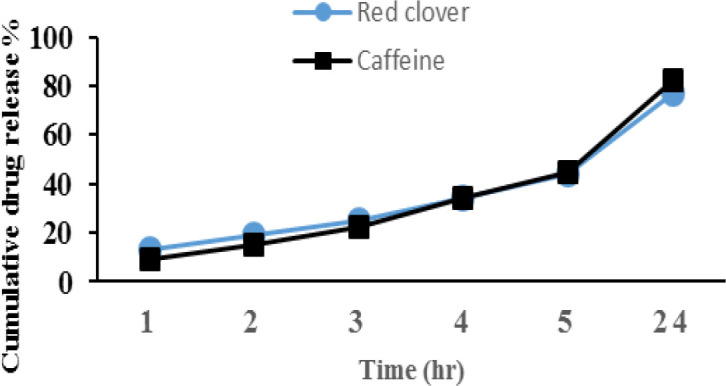
Percent of cumulative release of drugs. One milliliter of each formulation was transferred to a dialysis bag. A phosphate buffer (pH 6.3) was used as release medium. Samples were collected at 1, 2, 3, 4, 5 and 24 hr intervals and drug concentration was measured spectrophotometrically (mean±SD of 3 repetition at each time point).

Figure 1 shows the cumulative release of red clover and caffeine. As it is seen, after 24 hr, 77.6% of red clover and 76.95% of caffeine released in the medium. 

### Effect of different treatments on hair growth scores

**Figure 2:**
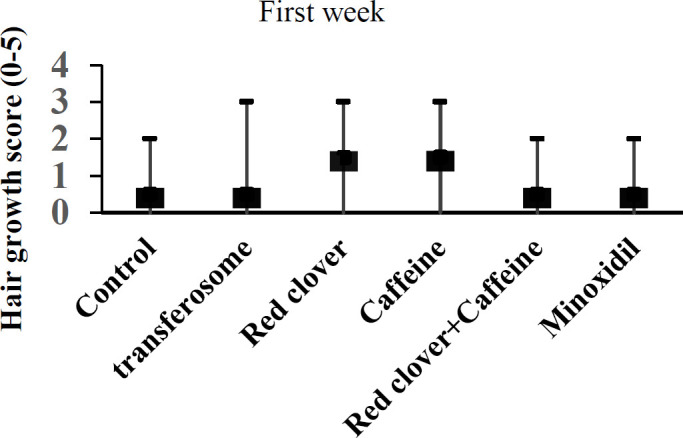
Effect of different treatments on the hair growth score after one week. Data shows median and range of growth score (n=6).

**Figure 3:**
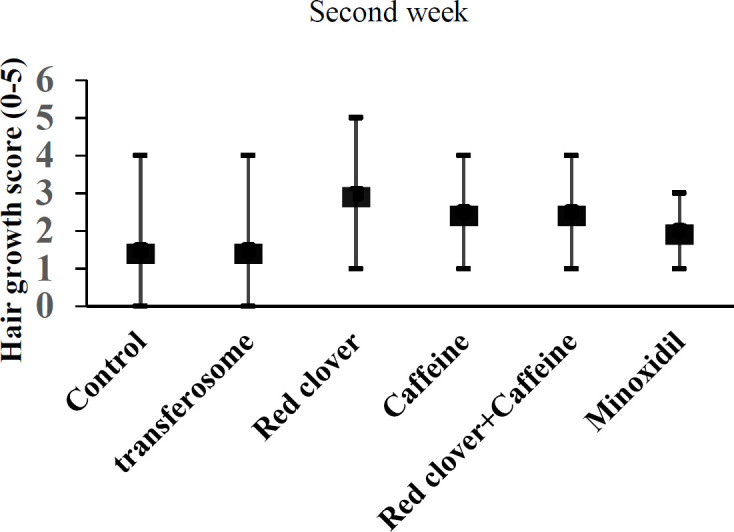
Effect of different treatments on the hair growth score after two weeks. Data shows median and range of growth score (n=6).

**Figure 4:**
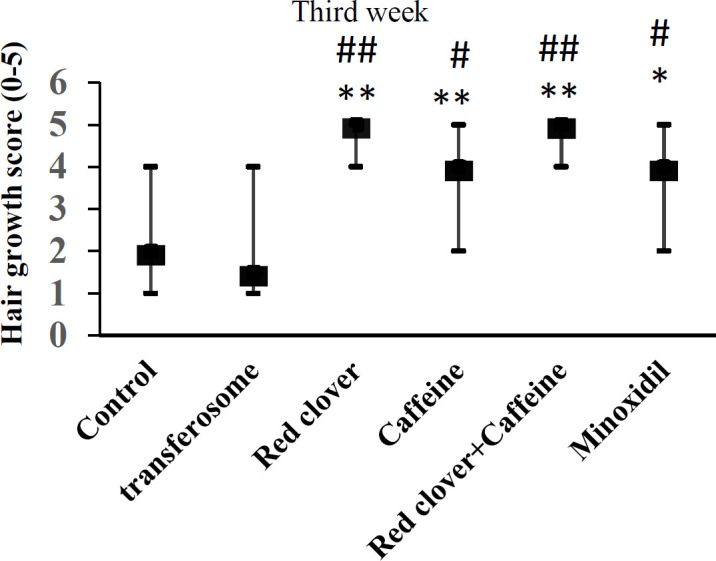
Effect of different treatments on the hair growth score after three weeks. Data shows median and range of growth score (n=6).*p<0.05 and **p<0.01 compared with the control group.#p<0.05 and ##p<0.01 compared with the transferosome base (Mann-Whitney U test).

Seven days treatment with red clover or caffeine alone increased the hair growth score but the effect was not significant (Figure 2). At the end of the second week, the hair growth scores in all drug-treated groups was greater than control and transferosome base but again the changes were not significant (Figure 3). After 21 days, the hair growth scores in all drug-treated groups were significantly different in comparison with control or transferosome groups (Figure 4).

**Figure 5:**
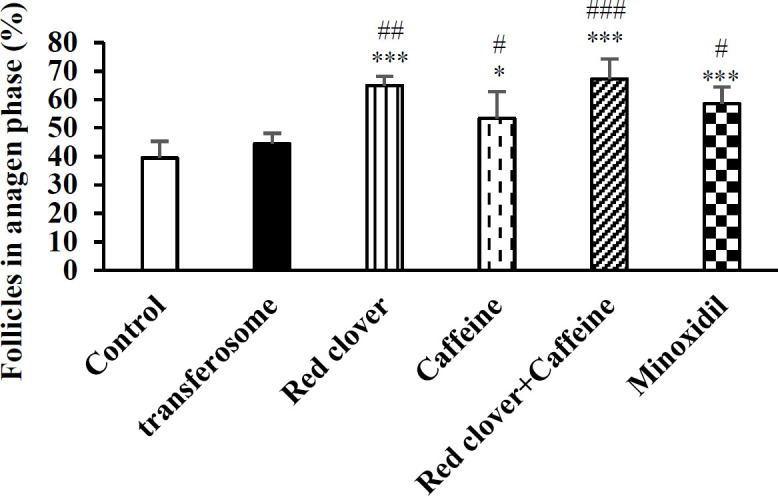
Effect of different treatments on percent of the follicles in the anagen phase. Data shows mean±SEM(n=6). *p<0.05 and ***p<0.001 compared with the control group. #p<0.05; ##p<0.01 and ###p<0.001 compared with the transferosome base. (one-way ANOVA followed by Scheffe post hoc)

The percent of follicles in the anagen phase for each treatment is depicted in Figure 5. Transferosome without drug is not significant from control. Red clover significantly (p<0.001) increased the percent of follicles in the anagen phase. Other treatments also produced significant effects. Combination of red clover and caffeine did not show an additive effect and the percent of follicles in the anagen phase was not different when compared with red clover or caffeine alone. Although the effect of red clover alone or its combination with caffeine was greater than minoxidil but statistical analysis did not reveal any significant difference. 

### Pathological findings

**Figure 6:**
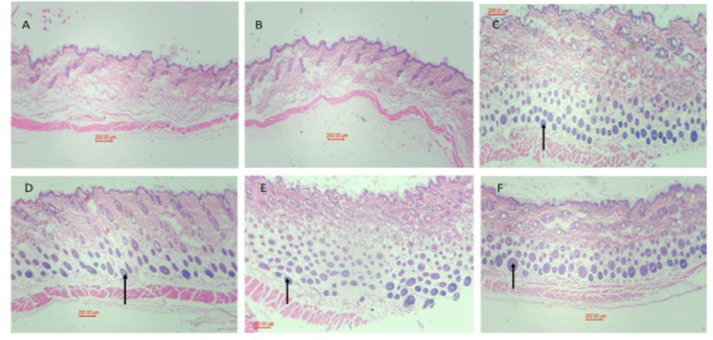
Pathological findings. 100 μl of formulations was applied topically on the shaved back skins of mice of all groups except group A (no treatment). Treatments continued once daily, 6 days a week for 3 weeks. Then animals were sacrificed and skin samples were prepared for pathological examination. A. Control; B. transferosome without drug; C. Red clover; D. Caffeine; E. Red clover + caffeine; F. Minoxidil. Arrows show the hair follicles.

All drug treatments increased the number of follicles as it is seen in Figure 6

## Discussion

In this study, transferosome formulations containing red clover, caffeine or both were prepared and the entrapment efficiency and *ex vivo* release was evaluated. Also, the formulations were applied topically on the hair-removed skin of mice for 3 weeks and hair growth was assessed. All formulations had an entrapment efficiency of more than 80% which indicate that drug loading occurred efficiently. Cumulative release from dialysis bag also showed that after 24 hr, releases of caffeine and red clover were 77.6% and 76.9%, respectively. The pharmacological findings showed that the treatments significantly increased hair growth scores and the percent of follicles in the anagen phase. Pathological examination also confirmed the above findings (Figure 6).

Transferosomes are known as special types of liposomes used for transdermal drug delivery. It has the ability to easily penetrate the stratum corneum. Advantages of transferosomes include enhanced penetration into skin, biocompatibility and biodegradability (Solanki et al., 2016). Minoxidil and finasteride as food and drug administration (FDA)-approved drugs for hair loss have been formulated as transferosomes in previous works (Ahmed and Rizq, 2018; Allam et al., 2022). Our results indicated that transferosomes can be 

used as a novel drug delivery system for topical application of red clover extract or caffeine. 

**Table 1:** Percent of entrapment efficiency of transferosomes

**Formulation**	**Caffeine**	**Red clover**	**Caffeine + Red clover**
Entrapment efficiency (EE%)	84.3	81.6	89.1

Previous studies have reported that red clover extract contains isoflavones like biochanin A and formononetin (Krenn et al., 2002). Hiipakka et al (2002) reported that these isoflavones inhibit 5-alpha reductase, the enzyme that has a key role in conversion of testosterone to dihydrotestosterone and is an important factor in male pattern hair fall (Hiipakka et al., 2002). Other studies have shown that a combination of acetyl tetrapeptide-3 and biochanin produce good results in both experimental and clinical studies on hair growth (Loing et al., 2013). In our study, red clover produced a considerable hair growth activity that was comparable to that of minoxidil. 

Several studies have focused on the hair growth effect of caffeine. Fischer et al. (2007) conducted an *ex vivo* study and demonstrated that caffeine (0.0001% and 0.005%) promoted the growth of hair follicles (Fischer et al., 2007). Also, in another study hair follicles obtained from the scalps of subjects who were treated with testosterone alone or testosterone plus caffeine and it was reported that caffeine increased the length of anagen phase and promoted hair follicle growth (Fischer et al., 2014). Dhurat et al (2017) conducted a randomized multicenter study and found that a topical liquid formulation of caffeine (0.2%) was as effective as minoxidil 5% solution (Dhurat et al., 2017). 

In agreement with above reports, our findings demonstrated hair growth promoting effect of caffeine in mice. In our study, one group of animals was treated with transferosomes containing both red clover and caffeine and the effect was not different from these agents alone indicating that additive or synergistic effect is not seen and it may be due to physicochemical interactions, common target mechanisms or the concentration of the drugs. 

In conclusion, transferosomes are promising formulations for delivery of red clover extract or caffeine to the scalp. The other finding is that combination of red clover extract and caffeine is not associated with additive of synergistic effect suggesting that in designing hair growth formulations caffeine or red clover alone is enough.
